# Real-time nowcasting and forecasting of COVID-19 dynamics in England: the first wave

**DOI:** 10.1098/rstb.2020.0279

**Published:** 2021-07-19

**Authors:** Paul Birrell, Joshua Blake, Edwin van Leeuwen, Nick Gent, Daniela De Angelis

**Affiliations:** ^1^ Public Health England, National Infection Service, 61 Colindale Avenue, London NW9 5HT, UK; ^2^ MRC Biostatistics Unit, University of Cambridge, East Forvie Site Building, Forvie Site, Robinson Way, Cambridge Biomedical Campus, Cambridge CB2 OSR, UK; ^3^ Public Health England, Emergency Response Department, Porton Down, SP4 0JG, UK

**Keywords:** real-time, dynamics, COVID-19, Bayesian, nowcasting, forecasting

## Abstract

England has been heavily affected by the SARS-CoV-2 pandemic, with severe ‘lockdown’ mitigation measures now gradually being lifted. The real-time pandemic monitoring presented here has contributed to the evidence informing this pandemic management throughout the first wave. Estimates on the 10 May showed lockdown had reduced transmission by 75%, the reproduction number falling from 2.6 to 0.61. This regionally varying impact was largest in London with a reduction of 81% (95% credible interval: 77–84%). Reproduction numbers have since then slowly increased, and on 19 June the probability of the epidemic growing was greater than 5% in two regions, South West and London. By this date, an estimated 8% of the population had been infected, with a higher proportion in London (17%). The infection-to-fatality ratio is 1.1% (0.9–1.4%) overall but 17% (14–22%) among the over-75s. This ongoing work continues to be key to quantifying any widespread resurgence, should accrued immunity and effective contact tracing be insufficient to preclude a second wave.

This article is part of the theme issue ‘Modelling that shaped the early COVID-19 pandemic response in the UK’.

## 1. Introduction

As of the 20 June more than eight million people have been reported as being infected by SARS-CoV-2 globally with over 450 000 confirmed deaths [1]. Having first been identified in Wuhan, SARS-CoV-2 rapidly spread to other Chinese provinces, Thailand, Japan and the Republic of Korea in the first three weeks of January 2020 [2], with an early incursion into Europe centred around clusters in Bavaria, Germany and Haute-Savoie, France, both linked to subsequent cases in Spain [3]. This international spread eventually led the World Health Organisation to declare a pandemic on 11 March 2020 [4]. In the UK, pandemic preparedness plans, developed since 2009 A/H1N1pdm, were rapidly activated with governmental emergency bodies and advisory groups convening before the end of January [5].

The UK response to the COVID-19 pandemic escalated from an initial containment effort to the suppression or ‘lockdown’ strategy introduced on the 23 March [6]. Over this period, through participation in governmental advisory groups, scientists from a number of research institutions fed into the pandemic decision-making processes through the work of the Scientific Pandemic Influenza Sub-Group on Modelling (SPI-M) [7]. This work has informed the various phases of the UK response, from constructing planning scenarios for the health system, to monitoring these scenarios through regular nowcasting of new infections and forecasting of severe disease and health service demand, see [8,9] and papers available from [7].

Here, we report the contribution of one of the participating groups, the Public Health England (PHE)/University of Cambridge modelling group. This collaboration, funded to develop modelling methodology for real-time pandemic influenza monitoring [10], was re-activated for the COVID-19 pandemic [11]. The age and spatially structured transmission model developed for influenza [12,13] has been adapted to the SARS-CoV-2 epidemiology and implemented through a Bayesian statistical analysis of pandemic surveillance data, incorporating knowledge on the natural history of infection from emerging literature. Throughout the first pandemic wave, the model has been continuously developed to include the information that progressively become available. Through this regular monitoring, we have been able to: anticipate and understand the impact of the lockdown; provide sequential updates of the pandemic transmission dynamics, by estimating the basic (*R*_0_) and effective (*R*_*t*_) reproduction numbers, (i.e. the average number of individuals infected by an infectious individual in a totally and partially susceptible population, respectively), and inform the gradual relaxation of the lockdown.

In what follows, we focus on our contribution to the monitoring of the first pandemic wave at specific dates spanning three key periods: pre-lockdown (before 23 March), lockdown (until 11 May); and two subsequent dates (3 and 19 June) that allowed the assessment of the gradual easing of the lockdown; we then conclude with an update over the summer and final consideration on the experience so far.

## 2. Data and methods

### (a) The transmission model

We model SARS-CoV-2 transmission through an age-stratified transmission models in each of the seven National Health Service (NHS) regions of England, where the regional epidemics share common parameters. Within each region, the infection dynamics are governed by a system of ordinary differential equations, discretized to give the following set of first-order difference equations:

2.1 $$\begin{array}{rl} S_{r,t_{k},i} & =S_{r,t_{k-1},i}(1-\lambda_{r,t_{k-1},i}\delta t)\\ E_{r,t_{k},i}^{1} & =E_{r,t_{k-1},i}^{1}\left(1-\frac{2\delta t}{d_{L}}\right)+S_{r,t_{k-1},i}\lambda_{r,t_{k-1},i}\delta t\\ E_{r,t_{k},i}^{2} & =E_{r,t_{k-1},i}^{2}\left(1-\frac{2\delta t}{d_{L}}\right)+E_{r,t_{k-1},i}^{1}\frac{2\delta t}{d_{L}}\\ I_{r,t_{k},i}^{1} & =I_{r,t_{k-1},i}^{1}\left(1-\frac{2\delta t}{d_{I}}\right)+E_{r,t_{k-1},i}^{2}\frac{2\delta t}{d_{L}}\\ \mathrm{and}\;I_{r,t_{k},i}^{2} & =I_{r,t_{k-1},i}^{2}\left(1-\frac{2\delta t}{d_{I}}\right)+I_{r,t_{k-1},i}^{1}\frac{2\delta t}{d_{I}} \end{array}\}$$

where: $S_{r,t_{k},i}$, $E_{r,t_{k},i}^{l}$, $I_{r,t_{k},i}^{l},l=1,2$ represent the time *t*_*k*_, *k* = 1, …, *K*, partitioning of the population of individuals in a region *r*, *r* = 1, …, *n*_*r*_, in age-group *i*, *i* = 1, …, *n*_*A*_, into *S* (susceptible), *E* (exposed) and *I* (infectious) disease states. The mean latent and infectious periods are *d*_*L*_ and *d*_*I*_, respectively; and $\lambda_{r,t_{k},i}$ is the time- and age-varying rate with which susceptible individuals become infected. Note that two *E* and *I* states are specified to make the model more flexible by allowing Gamma distributed times in the each of these disease states. Time steps of *δt* = 0.5 days are chosen to be sufficiently small relative to the anticipated latent and infectious periods. New infections are generated as

2.2 $$\Delta_{r,t_{k},i}^{\mathrm{infec}}=S_{r,t_{k},i}p_{r,t_{k},i}^{\lambda },$$

where

2.3 $$p_{r,t_{k},i}^{\lambda }=\left(1-\prod_{\;j=1}^{n_{A}}\left[{\left(1-b_{r,ij}^{t_{k}}\right)}^{I_{r,t_{k},j}^{1}+I_{r,t_{k},j}^{2}}\right]\right)\delta t\approx \lambda_{r,t_{k},i}\delta t.$$

Here, $b_{r,ij}^{t_{k}}$ is the probability of a susceptible individual in region *r* of age group *i* being infected by an infectious individual in age group *j* at time *t*_*k*_. It is a function of:

(i) a set of time-varying contact matrices $C^{t_{k}}=\{C_{ij}^{t_{k}}\}$, with $\left\{C_{ij}^{t_{k}}\right\}$ describing the expected number of contacts between individuals in strata *i* and *j* within a single time unit *t*_*k*_;
(ii) $M_{r}^{t_{k}}=\{M_{r,ij}^{t_{k}}\}$, a region-specific matrix, whose (*i*, *j*)th element gives the relative susceptibility of someone in age-group *i* to an infection from an infectious individual in age-group *j* assuming contact between the two. Many of the components of this matrix are assumed to be 1, but some are specified as unknown parameters *m*_*r*,*l*_, describing the relative susceptibility in the over-75s and the proportionate change in susceptibility for both under- and over-75s after the lockdown;
(iii) $\beta_{t_{k},r}$, a time-varying parameter encapsulating further temporal fluctuation in transmission that applies to all ages (see the electronic supplementary material, equation (9));
(iv) *R*_0,*r*_, the initial reproduction numbers for the pandemic in each region at time *t*_0_. This is a function of two unknown parameters, a region-specific growth rate, *ψ*_*r*_ and *d*_*I*_; and
(v) $R_{0,r}^{*}$, the dominant eigenvalues of the initial next-generation matrices, $\Lambda_{0,r}$:
  2.4 $$\Lambda_{0,r,ij}=N_{r,i}{\tilde{C}}_{r,ij}^{t_{0}}d_{I},$$
  where *N*_*r*,*i*_ is the population size in region *r* and age-group *i*; and ${\tilde{C}}_{r}^{t_{k}}$ are a set of matrices defined by
  $${\tilde{C}}_{r}^{t_{k}}=C^{t_{k}}⊙M_{r}^{t_{k}}$$
  with the ⊙ notation indicating element-wise multiplication, such that A=B⊙C if *A*_*ij*_ = *B*_*ij*_*C*_*ij*_.

The $C^{t_{k}}$ matrices encode the information about contact rates between different age groups derived from the POLYMOD study [14], Google mobility and the time-use survey. The $M_{r}^{t_{k}}$ matrices capture any mis-specification of these matrices in terms of the changing pattern of infection between the age groups, whereas the $\beta_{t_{k}}$ parameters account for mis-specification of the changing scale of transmission over time as described by the matrices.

The general expression of $b_{r,ij}^{t_{k}}$ is:

2.5 $$b_{r,ij}^{t_{k}}=\frac{\beta_{t_{k},r}R_{0,r}}{R_{0,r}^{*}}{\tilde{C}}_{r,ij}^{t_{k}};$$

although analyses in the earliest periods involve a simplified version of equation (2.5) (see the electronic supplementary material) for details on the specific expressions).

The transmission dynamics described above depend on the parameters *d*_*I*_ and *d*_*L*_ in equation (2.1); the parameters specifying $b_{r,ij}^{t_{k}}$; and the initial conditions of the system, which can also be expressed as parameters (see the electronic supplementary material, table S3). These unknown parameters are either fixed to values derived from the literature or estimated from the combination of different data sources linked to the latent transmission process through observational models (see §2c).

### (b) Data sources

**Figure 1.**
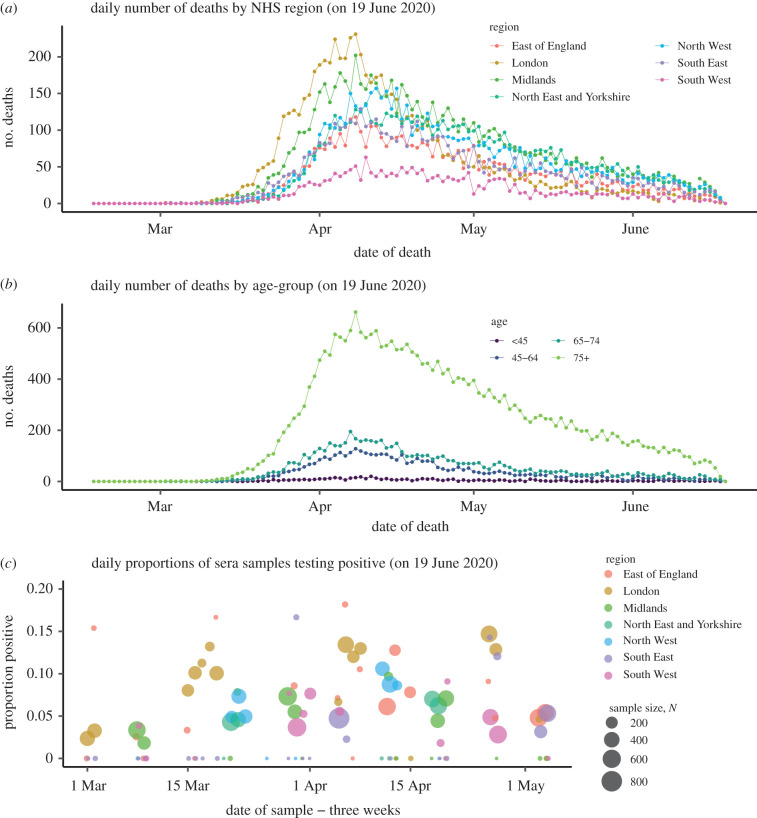
(*a*) Data on deaths by region, and (*b*) age; and (*c*) serological positivity by region and sampling date.

The surveillance data used are age- and region-specific counts of deaths of people with a laboratory-confirmed COVID-19 diagnosis (figure 1*a*,*b*). From 21 April onwards, weekly batches of serological data, indicating the fraction of the population carrying COVID-19 antibodies, from NHS Blood and Transplant (NHSBT) samples (figure 1*c*) [15] have also been included.

The Wuhan outbreak additionally provides information on epidemiological parameters: the duration of infectiousness, the mean time from infection to symptom onset [16]; the probability of dying given infection and the mean time from symptoms onset to death [17]. Central to age-specific epidemic modelling, contact patterns between age groups have been derived from the POLYMOD study [14] stratified by setting (school, workplace, leisure etc), with these matrices sequentially updated using the Google mobility study and the UK time-use survey [18], to quantify the change in population mobility and access to these contact settings over time (see the electronic supplementary material, Contact matrices).

### (c) Parameter estimation

Estimation is carried out in a Bayesian framework, and parameter estimates (and attached uncertainty) are based on the joint posterior distribution of all the parameters, derived by combining prior knowledge on model parameters with the likelihood of the data. The likelihood for each data source is constructed by treating the data as imperfect observations of modelled quantities with error taking an appropriate statistical distribution. For example, the daily number of deaths are derived from the model by assuming that each day a fraction of new infections will die and specifying a time from infection to death. The observed deaths then follow a negative binomial distribution with mean the model-derived deaths and an appropriate (unknown) dispersion parameter. Similarly, serological data are assumed to be binomially distributed with a mean related to the susceptible fraction of the population.

The total number of unknown parameters to be estimated includes the transmission dynamics parameters, the natural history parameters (e.g. the probability of dying given infection) and the parameters of the observational models (see the electronic supplementary material, Inference section and table S3 for more details).

The posterior distribution cannot be evaluated analytically and is estimated using Markov chain Monte Carlo (MCMC) [12,13]. The most recent analysis featured in this paper was based on 900 000 iterations, with an initial adaptive phase of 45 000 iterations within a burn-in period of 90 000 iterations. Parameter estimates are based on the full sample following burn-in thinned to retain every 125 iterations, and projections are based on a sample thinned to every 250 iterations. All central estimates are pointwise medians of quantities calculated on the basis of this sample, and uncertainty is expressed through 95% credible intervals (CrI) derived from the 2.5% and 97.5% quantiles. The implementing code (in C++) and model framework are available from https://gitlab.com/pjbirrell/real-time-mcmc/-/tree/COVID.

## 3. Results

### (a) Pre-lockdown

After initial attempts to contain the pandemic through trace and test strategies [19] and to mitigate the burden on the NHS through combinations of non-pharmaceutical interventions (e.g. case isolation, restrictions on foreign travel, shielding of vulnerable groups, cancellation of mass gatherings), the pressing question became: what level of stringent social distancing measures would be necessary to suppress transmission? At this stage, infection was not sufficiently widespread in each of the seven NHS regions for the data to inform a fully stratified model, by this time there had only been four deaths in the whole of the South West. Therefore, no age structure was used and the country was stratified into two regions: London, where the number of deaths was significantly higher (figure 1*a*), and outside London. Assuming a pandemic intervention is imposed on 23 March, the model was fitted to data on COVID-19 confirmed deaths to 15 March, and then used to project epidemic curves forward a further eight weeks. These projections assumed differing reductions in contact rates (24%, 48%, 64%) (see the electronic supplementary material, equation (8)) and consequently transmission. The suggested reductions were achieved by removing ‘school’ and ‘leisure’ based contacts from contact surveys in the socialmixr package for statistical software R [20].

**Figure 2.**
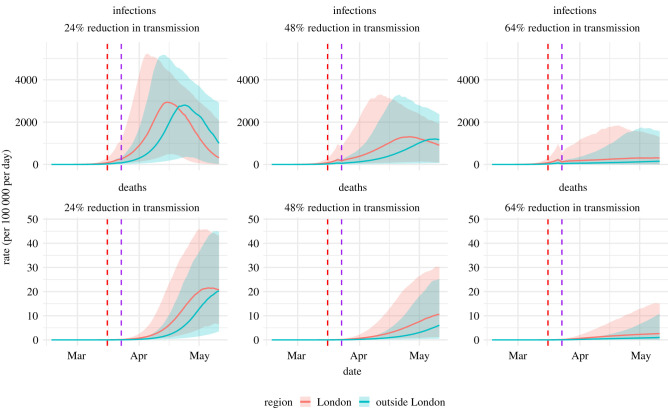
Estimated and projected COVID-19 infections and deaths by efficacy of social restriction measures.

Figure 2 shows the projections for different levels of this reduction. The dashed red vertical line shows the date of the most recent data included in the analysis and the dashed purple line represents the timing of the intervention. Each column shows the projected epidemic infection and death rates under three assumed intervention impacts.

The most optimistic scenario in figure 2 corresponded to an immediate 64% reduction in transmission. Under this assumption, *R*_*t*_ was estimated to be 1.2 (95% CrI: 0.83–2.1) in London and 1.2 (95% CrI: 0.84–2.2) elsewhere. In this scenario, the probability that the imposed measures were successful in reducing *R*_*t*_ to the threshold of 1 required for declining transmission was only 19% and 17% in the two regions, respectively. To be 95% certain that the intervention would lead to a sustained decline in infection, the intervention would need to induce an 81% reduction in transmission. Such a reduction could only be achieved through the implementation of extreme mitigation measures.

### (b) The lockdown period

The number of deaths continued to rise until 8 April, particularly in older age-groups (figure 1*b*), permitting stratification of the model by both age (eight groups) and region (seven NHS regions). Also, new information from serological studies (figure 1*c*) started to become available and weekly data, downloaded from the Google mobility survey, could be used to update contact matrices.

A rhythm for pandemic monitoring was established. The model was run daily, with results feeding into local planning tools as well as the SPI-M consensus view on the state of the pandemic, and with periodic publication of web-reports summarizing the latest results [21]. These outputs included a number of key indicators: regional estimates of *R*_*t*_ and epidemic growth rates *r*, indicating whether transmission is increasing (*R*_*t*_ > 1) and the rate at which it is increasing [22]; region and age-specific attack rates (AR) (i.e. the proportion of the population already infected); and predictions of the burden owing to mortality, both in terms of age-specific infection-fatality ratios (IFR) and number of COVID-19 deaths. Public attention has focused on *R*_*t*_ as a headline figure for the state of the pandemic, but a more complete assessment requires all these indicators. Table 1 presents estimates of a selection of these indicators, giving snapshots of the pandemic state at the three chosen times.

**Table 1.** Table of estimates (with 95% credible intervals attached) for key epidemic parameters and derived quantities.

analysis	region	*R_t_*	*r*	infections	AR	IFR (overall)	IFR (75+)
**date**							
**10th May**	East	0.71	−0.07	1130	10%	—	—
		(0.68, 0.74)	(−0.08, −0.06)	(758, 1660)	(8%, 13%)	—	—
	London	0.40	−0.18	24	20%	—	—
		(0.36, 0.43)	(−0.20, −0.16)	(10, 53)	(16%, 26%)	—	—
	Mids	0.68	−0.08	1490	11%	—	—
		(0.65, 0.71)	(−0.08, −0.07)	(1080, 2040)	(9%, 15%)	—	—
	NE&Y	0.80	−0.05	4320	11%	—	—
		(0.76, 0.83)	(−0.05, −0.04)	(3230, 5650)	(8%, 14%)	—	—
	North West	0.73	−0.06	2380	14%	—	—
		(0.70, 0.76)	(−0.07, −0.06)	(1750, 3160)	(11%, 18%)	—	—
	South East	0.71	−0.07	1260	8%	—	—
		(0.68, 0.74)	(−0.08, −0.06)	(855, 1810)	(6%, 11%)	—	—
	South West	0.76	−0.06	739	5%	—	—
		(0.72, 0.80)	(−0.07, −0.05)	(438, 1200)	(4%, 6%)	—	—
	**England**	**0.75**	**−0.06**	**11400**	**12%**	**0.6%**	**16%**
		**(0.72, 0.77)**	**(−0.06, −0.05)**	**(9150, 14200)**	**(9%, 15%)**	**(0.5%, 0.8%)**	**(12%, 21%)**
**3rd June**	East	0.94	−0.01	1660	9%	—	—
		(0.73, 1.14)	(−0.06, 0.03)	(502, 4610)	(8%, 10%)	—	—
	London	0.95	−0.01	1310	17%	—	—
		(0.72, 1.20)	(−0.07, 0.04)	(247, 4670)	(15%, 19%)	—	—
	Mids	0.90	−0.02	2460	10%	—	—
		(0.73, 1.07)	(−0.07, 0.01)	(809, 6070)	(9%, 11%)	—	—
	NE&Y	0.89	−0.02	2450	9%	—	—
		(0.75, 1.04)	(−0.07, 0.01)	(865, 5870)	(8%, 11%)	—	—
	North West	1.01	0.0	4170	12%	—	—
		(0.83, 1.18)	(−0.04, 0.04)	(1580, 9840)	(10%, 14%)	—	—
	South East	0.97	−0.01	2420	7%	—	—
		(0.78, 1.17)	(−0.05, −0.03)	(782, 6040)	(6%, 8%)	—	—
	South West	1.00	0.0	778	4%	—	—
		(0.77, 1.29)	(−0.06, 0.06)	(162, 3080)	(3%, 5%)	—	—
	**England**	**0.99**	**0.0**	**16700**	**10%**	**0.9%**	**23%**
		**(0.91, 1.09)**	**(−0.02, 0.02)**	**(10700, 25300)**	**(9%, 11%)**	**(0.8%, 1.0%)**	**(20%, 27%)**
**19th June**	East	0.80	−0.05	292	7%	—	—
		(0.60, 1.01)	(−0.10, 0.00)	(60, 1050)	(6%, 8%)	—	—
	London	0.87	−0.03	837	17%	—	—
		(0.67, 1.12)	(−0.08, 0.02)	(159, 3070)	(15%, 18%)	—	—
	Mids	0.82	−0.04	709	8%	—	—
		(0.64, 1.01)	(−0.09, 0.00)	(189, 2040)	(7%, 9%)	—	—
	NE&Y	0.76	−0.05	351	7%	—	—
		(0.58, 0.95)	(−0.11, −0.01)	(84, 1110)	(6%, 8%)	—	—
	North West	0.84	−0.04	872	10%	—	—
		(0.69, 1.02)	(−0.08, 0.00)	(255, 2460)	(9%, 11%)	—	—
	South East	0.77	−0.05	342	6%	—	—
		(0.59, 0.96)	(−0.10, −0.01)	(79, 1120)	(5%, 6%)	—	—
	South West	0.94	−0.01	312	3%	—	—
		(0.69, 1.21)	(−0.07, 0.04)	(60, 1180)	(3%, 4%)	—	—
	**England**	**0.88**	**−0.03**	**4260**	**8%**	**1.1%**	**17%**
		**(0.79, 1.01)**	**(−0.05, 0.00)**	**(2370, 7290)**	**(8%, 9%)**	**(0.9%, 1.4%)**	**(14%, 22%)**

**Figure 3.**
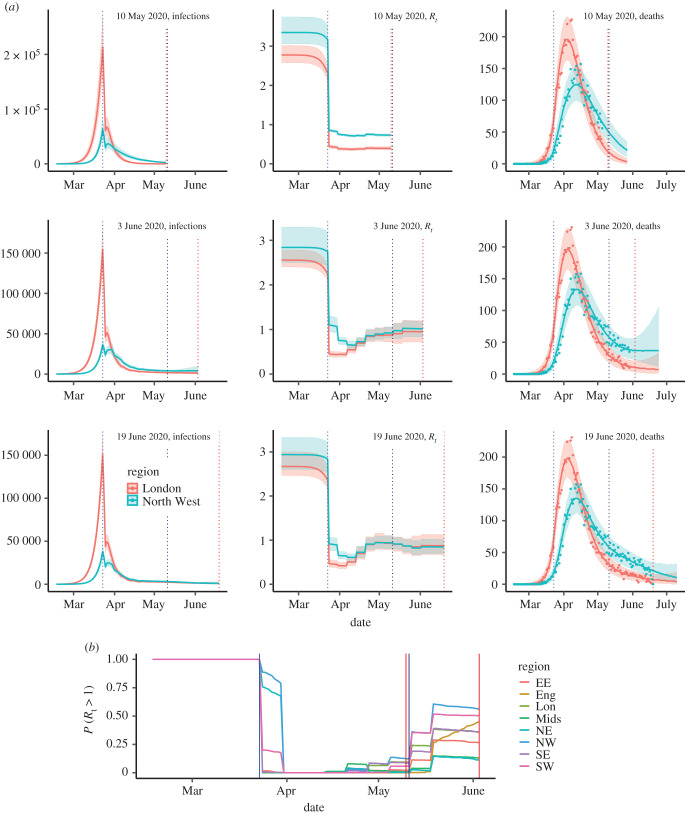
(*a*) Sequentially obtained estimates infection trajectories; *R*_*t*_; and three-week forecast of the number of deaths by date of analysis (rows). (*b*) Probability that *R*_*t*_ exceeds 1 as of 3 June, by region. See the electronic supplementary material, figure S3 for 10 May and 19 June. In both plots, vertical blue lines indicate the timings of significant policy changes, vertical red lines indicate the time of analysis.

The 10 May section of the table shows the success of the lockdown at curtailing transmission: the *R*_*t*_ in England is now estimated to be 0.75 (95% CrI: 0.72–0.77) having dropped from 2.6 (95% CrI: 2.4–2.9) to 0.61 (95% CrI: 0.57–0.67) at the time of the lockdown, a reduction of 75% (95% CrI: 73–77%), in line with the anticipation of the pre-lockdown modelling. The ‘growth’ rate for England indicates the daily number of infections were halving every log (2)/*r* = 11.5 days. London stands out as the region with the highest estimated attack rate (20% of people infected, CrI: 16–26%), largely owing to pre-lockdown levels of infection; the largest reduction in transmission, a drop of 81% (95% CrI: 77–84%) to *R*_*t*_ = 0.4, 95% CrI: 0.36–0.43, and the steepest rates of decline in both the number of infections (halving every 4 days) and the observed fall in the number of deaths (figure 1*a*). The temporal patterns in infection are disrupted at the lockdown date, with the top row of figure 3*a* illustrating the size of this effect for both the London and North West regions, alongside the estimated *R*_*t*_.

Launched on the 10 May, the UK COVID-19 4-level Alert System, coordinated by the newly established Joint Biosecurity Centre (JBC) and based on both estimated *R*_*t*_ and current infections, highlighted the key role of these indicators [6] in guiding the relaxation of lockdown measures without re-igniting transmission.

### (c) Lockdown relaxation

The first tranche of relaxations were announced on the 10 May. From this time, accounting for likely changes in behaviour became crucial. In addition, it was clear, from the over-precision of the estimates of both incidence and *R*_*t*_ in the top row of figure 3*a*, that the model required a greater flexibility to capture such changing behaviours. From an appropriately adapted model (see the electronic supplementary material, Transmission model for details), we estimated that at the 3 June, *R*_*t*_ for England reached 0.99 (95% CrI: 0.91–1.09), with the probability of exceeding the value of 1 rapidly increasing over time (figure 3*b*). This figure masks regional heterogeneity in transmission. The North West and the South West were characterized by *R*_*t*_ values above 1 (with a probability *R*_*t*_ > 1 of over 50%, figure 3*b*) and growth rate estimates encompassing positive values. For the North West, we estimated 4170 (95% CrI: 1580–9840) daily infections, the highest number in the country (table 1). The step changes in the plots of *R*_*t*_ over time in figure 3 for 10 May are entirely owing to changes in the Google mobility matrices. Looking at the equivalent plot for the 3 June analysis, over the same interval, the step changes are larger. This difference in the level of fluctuation over time suggests that the increases in *R*_*t*_ are too large to be solely attributed to mobility-driven changes in the contact matrices. Furthermore, for the North West, the drop in *R*_*t*_ around the lockdown is not as sharp as in London, but rather staggered over three weeks. This might suggest a different response to the lockdown in these two regions, which we had previously not been able to identify. The estimated steady resurgence of *R*_*t*_ in the North West ultimately led to a policy change, delaying the staged re-opening of schools [23].

Continuing to monitor the pandemic evolution in the post-lockdown era, we adapted the model to incorporate new evidence on differential susceptibility to infection by age [24] (see the electronic supplementary material, around equation (10)). Results from 19 June data (table 1 and figure 3*a*) show lower estimates for *R*_*t*_, negative growth rates and the estimated number of infections in England decreasing to 4300 (95% CrI: 2400–7300). There is still regional heterogeneity, with two regions for which the CrIs for *R*_*t*_ exclude 1 (North East & Yorkshire, and the South East); and the probability that the epidemic is growing is 30% in the South West and below 15% in each of the other regions (electronic supplementary material, figure S3*b*). Throughout, we have been estimating age-specific infection-fatality ratios (see table 1; electronic supplementary material, table S4). Allowing for differential age susceptibility, the age-specific estimates of the infection-fatality ratio fall to 17% (95% CrI: 14–22%) in the over-75s (from 23%, 95% CrI: 20–27%) with a rise to 2.9% in the 65–74s, (see the electronic supplementary material, table S4) and to 1.1% (95% CrI: 0.9–1.4%) from 0.9% (95% CrI: 0.8–1.0%) overall. These less severe estimates (in comparison to the 3 June analysis) led to the UK Chief Medical Officers agreeing with a JBC recommendation that the alert level should be downgraded to level three.

### (d) The summer

Since the 19 of June the weekly updating has continued. Subsequent estimates of the IFR have varied in the region of 0.9 to 1.4% overall and 14 to 19% in the over-75s. *R*_*t*_ has approached the value of 1 in most regions without exceeding it, which, together with a decreasing number of daily infections indicates an epidemic still in decline, although local outbreaks are being increasingly detected [25]. The estimates presented here are consistent with the SPI-M consensus on the values of *R*_*t*_ both nationally and regionally [26]. Incidence estimates can be contrasted with estimates from community cross-sectional studies. In a report of the 9 July, the Office for National Statistics (ONS) estimates 1700 (range 700–3700) new daily infections over the two weeks leading up to 4 July [27], while the COVID Symptom Study app developed by ZOE Global Ltd [28] reports an average 1470 infections per day over a similar period. In our work, incidence ranges from a central estimate of 4200 daily infections down to 3500 over the period. These are not incongruous to the ZOE app estimates, which only refer to symptomatic infection and require some scaling to derive an estimate for total infections. The ONS estimate is likely to be an underestimate as the survey does not include individuals in institutionalized settings, e.g. care homes, where incidence may be far higher than in the community. The degree to which this is an underestimate, however, is unclear.

The ONS also reports [29] that 6.3% (95% CrI: 4.7–8.1%) of individuals showed the presence of antibodies to the COVID-19 in blood sera samples (as of 19 June), a little lower than the 8% estimated attack rate for England. This discrepancy may well be owing to the timing of the samples, due to the waning of the antibody response over time. Estimates of attack rates (table 1) show that our belief on the proportion of the population that has been infected is being revised downwards at each sequential analysis. This, together with the emerging evidence of waning immunity [30], paint a muddied picture in terms of the potential for a population-level resurgence in infection.

On the 4 July, there was a significant step change in the gradual relaxation of pandemic mitigation measures as leisure facilities, tourist attractions, pubs and cafes all became accessible to the public once again [31]. The impact of this has been progressively observed throughout the summer via increasing episodes of localized outbreaks, with the public health emphasis moving from national and regional monitoring to the identification of local hot-spots and the imposition of local measures.

The estimated *R*_*t*_ have oscillated around 1 in a number of regions, although they become progressively more uncertain as the number of deaths have continued to decrease. The incidence of new infections has also slowly decreased from around 4000 new infections per day at the beginning of July (see the electronic supplementary material, figure S3). By the end of August, infection levels are relatively low at 2400 (95% CrI: 1300–4500) total daily infections, consistent with the ZOE app and ONS estimates of 1980 and 2200 (95% CrI: 1100–3800), respectively; and 8% (95% CrI: 7–13%) of the population had been infected in England, 16% (95% CrI: 13–24%) in London and 10% (95% CrI: 8–15%) in the North West.

In the first two weeks of September, following patterns already observed in other European countries, signs of resurgence are accumulating: an increasing number of regions are reporting multiple localized outbreaks; incidence estimates from ONS and the ZOE app have risen to 3200 and 3800 new daily infections, respectively; and results from ONS and REACT (https://www.imperial.ac.uk/medicine/research-and-impact/groups/react-study/) studies show increased COVID-19 prevalence.

### (e) Final considerations

Tracking the pandemic throughout its first wave has been an incredibly interesting and challenging experience. The pace of the updates required by SPI-M as data accumulated has been fast. We have learned to interpret newly emerging information swiftly, to adapt the model’s structure rapidly and to tailor the fitting algorithm promptly. We have developed a monitoring tool and progressively adapted it to deal with emerging challenges. Inevitably, the rate at which results had to be provided demanded compromises. We have not been able yet, for instance, to understand and incorporate data on hospitalizations, nor account properly for waning antibody responses, limiting the use of the NHSBT data. Also, the running times of the naive MCMC algorithm we used in the fitting of the model is $O(K^{2})$, and, as data continue to accumulate, our ability to produce results in a timely manner is going to be reduced.

As the pandemic progresses, short-term and longer-term model developments will be required. We can anticipate the need to specify different susceptibilities to infection, time-varying IFRs, seasonality in transmission and mortality, and, eventually, vaccine efficacy. These will all be required in the model at some stage, with possible heterogeneity across age-groups and regions. When to adapt the model to increase the complexity with which we handle each of these aspects will be a key modelling question, particularly if it involves a trade-off with model running times. Beyond this pandemic, methods for automatically handling the increasing complexities as data accumulate should be developed to ease the burden of real-time in-pandemic model development. This should motivate work for years to come.

## 4. In context

As hinted at in the various parts of the paper, this work has contributed, and is still contributing, to the SPI-M consensus on the evolution of the pandemic. Initially, we provided evidence of the likely effect of social restrictions on infections, deaths and hospitalizations, showing that a reduction in COVID-19 burden could only be achieved through the implementation of extreme measures (https://www.gov.uk/government/publications/phe-real-time-model-initial-results-1-march-2020). We then regularly produced updates of the current state of the pandemic as summarized by estimates of *R* values, infection growth rates (and doubling times) as well as infection incidence and prevalence, for England and the English NHS regions. The model has also been (and is being) used to derive medium term predictions (six weeks) of the number of deaths, again both nationally and by English regions. Our estimates of the IFR have been employed to produce worse case scenarios for mortality [32]; and, less public, but equally important, our regular outputs have been used extensively across PHE and JBC, also informing PHE regional resource planning and the scheduling of paediatric elective surgeries. Finally, our regular publications https://www.mrc-bsu.cam.ac.uk/tackling-covid-19/nowcasting-and-forecasting-of-covid-19/ have attracted significant media interest. We were, controversially, the first to highlight the regional heterogeneity in transmission, leading to local changes (e.g. https://www.theguardian.com/education/2020/jun/06/all) in policy.

We started producing regular modelling outputs, albeit from a very simple model, at the end of March 2020. However, we were only able to make our work publicly available on *MedRxiv* (https://www.medrxiv.org/content/10.1101/2020.08.24.20180737v1) in the summer. Like with other groups, the pressure to provide results at pace to inform the governmental response, while allowing adaptation of the model to account for accumulating data and the imposition of pandemic interventions, did not leave much time to the production of academic outputs.

We can now incorporate data on prevalence from the ONS Infection Survey; we have allowed for changes in IFR, following recent publications (https://arxiv.org/ftp/arxiv/papers/2103/2103.04867.pdf); and introduced vaccination. All of these have required a number of model developments. We look forward to publishing our methods and results for the second wave and beyond.

## Data accessibility

Mobility data and time use data are freely available. The death data used are daily totals aggregated to the seven NHS Regions and age-groups of broad width. These data are available upon the signing of a data-sharing agreement with Public Health England (contact wncov.modelling@phe.gov.uk).
